# Lung metastases from melanoma: when is surgical treatment warranted?

**DOI:** 10.1054/bjoc.2000.1335

**Published:** 2000-08-16

**Authors:** F Leo, L Cagini, P Rocmans, M Cappello, A N Van Geel, G Maggi, P Goldstraw, U Pastorino

**Affiliations:** Department of Thoracic Surgery, European Institute of Oncology, Via Ripamonti 435, Milan, 20141, Italy; Department of Surgery, University of Perugia, Italy; Department of Thoracic Surgery, Hospital Erasme, Brussels, Belgium; Department of Surgical Oncology, Dr D den Hoed Cancer Center, Rotterdam, Netherlands; Department of Thoracic Surgery, University of Torino, Italy; Department of Thoracic Surgery, Royal Brompton Hospital, London, UK

**Keywords:** pulmonary metastatic melanoma, metastasectomy

## Abstract

Surgical treatment of lung metastases from melanoma is highly controversial as the expected outcome is much poorer than for other primary tumours and a reliable system for selecting patients is lacking. This study evaluated the long-term results of lung metastasectomy for melanoma, with the aim of defining a subset of patients with better prognosis. By reviewing the data of the International Registry of Lung Metastases (IRLM), we identified 328 patients who underwent lung metastasectomy for melanoma in the period 1945–1995. Survival was calculated by Kaplan–Meier estimate, using log-rank test and Cox regression model for statistical analysis. After complete pulmonary metastasectomy (282 patients) the 5- and 10-year survival was 22% and 16%, respectively. In this group of patients, a time to pulmonary metastases (TPM) shorter than 36 months or the presence of multiple metastases were independent unfavourable prognostic factors. There were no long-term survivors after incomplete resection (46 patients, *P*< 0.01). Using the IRLM grouping system, patients without risk factors (TPM > 36 months and single lesion) experienced the best survival (29% at 5 years), followed by those with one risk factor only (20% at 5 years). On the other hand, those with two risk factors or incomplete resection showed a significantly poorer survival (7% and 0% at 5 years). Surgery plays an important role in carefully selected cases of pulmonary metastatic melanoma. The prognostic grouping system proposed by the International Registry of Lung Metastases provides a simple and effective method for improving the selection of surgical candidates. © 2000 Cancer Research Campaign


British Journal of Cancer (2000) 83(5), 569–572
© 2000 Cancer Research Campaign
doi: 10.1054/ bjoc.2000.1335, available online at http://www.idealibrary.com on IDEAL®

### Lung metastases from melanoma: when is surgical treatment warranted?

**F Leo[1], L Cagini[2], P Rocmans[3], M Cappello[3], AN Van Geel[4], G Maggi[5], P Goldstraw[6] and U Pastorino[1]**

[1]Department of Thoracic Surgery, European Institute of Oncology, Via Ripamonti 435, 20141 Milan, Italy; [2]Department of Surgery, University of Perugia, Italy; [3]Department of Thoracic Surgery, Hospital Erasme, Brussels, Belgium; [4]Department of Surgical Oncology, Dr D den Hoed Cancer Center, Rotterdam, Netherlands; [5]Department of Thoracic Surgery, University of Torino, Italy; [6]Department of Thoracic Surgery, Royal Brompton Hospital, London, UK

**Summary** Surgical treatment of lung metastases from melanoma is highly controversial as the expected outcome is much poorer than for other primary tumours and a reliable system for selecting patients is lacking. This study evaluated the long-term results of lung metastasectomy for melanoma, with the aim of defining a subset of patients with better prognosis. By reviewing the data of the International Registry of Lung Metastases (IRLM), we identified 328 patients who underwent lung metastasectomy for melanoma in the period 1945–1995. Survival was calculated by Kaplan–Meier estimate, using log-rank test and Cox regression model for statistical analysis. After complete pulmonary metastasectomy (282 patients) the 5- and 10-year survival was 22% and 16%, respectively. In this group of patients, a time to pulmonary metastases (TPM) shorter than 36 months or the presence of multiple metastases were independent unfavourable prognostic factors. There were no long-term survivors after incomplete resection (46 patients, $P < 0.01$). Using the IRLM grouping system, patients without risk factors (TPM > 36 months and single lesion) experienced the best survival (29% at 5 years), followed by those with one risk factor only (20% at 5 years). On the other hand, those with two risk factors or incomplete resection showed a significantly poorer survival (7% and 0% at 5 years). Surgery plays an important role in carefully selected cases of pulmonary metastatic melanoma. The prognostic grouping system proposed by the International Registry of Lung Metastases provides a simple and effective method for improving the selection of surgical candidates. © 2000 Cancer Research Campaign

**Keywords:** pulmonary metastatic melanoma; metastasectomy

Prognosis of patients with metastatic melanoma is poor (Ketcham and Balch, 1985; Tafra et al, 1995), with an annual risk of death of about 20% during the first 3 years (Slingluff et al, 1992). The second most common site for metastatic spread is the lung (Balch and Milton, 1985), and the annual probability of developing pulmonary metastases increases progressively from 10% at 5 years to 17% at 15 years. Overall long-term survival for such patients is poor, with only 4% of patients alive at 4 years (Harpole et al, 1992).

As effective chemotherapy for metastatic melanoma is not available, surgery can represent the only prospect of cure for highly selected patients (Wong et al, 1993; Karakousis et al, 1994). However only 10–12% of cases are suitable for surgery with curative intent (Cahan, 1973; Mountain et al, 1984; Thayer and Overholt, 1985; Pogrebniak et al, 1988; Gorenstein et al, 1991). Moreover, surgical results are still controversial as the majority of published studies are based on a small number of cases with relatively short follow-up.

Two large series have already confirmed the prognostic value of factors such as radicality, number of resected metastases and disease-free interval (DFI) after pulmonary metastasectomy (Harpole et al, 1992; Tafra et al, 1995) but it remains unclear how these data can be used for preoperative patient selection.

*Received 15 February 1999*
*Revised 1 May 2000*
*Accepted 17 May 2000*

*Correspondence to:* F Leo

The purpose of this study is to evaluate the long-term results of surgery in melanoma pulmonary metastases from the International Registry of Lung Metastases (IRLM) data, with the aim of defining the subset of patients that really benefit from surgery.

### PATIENTS AND METHODS

Of the 5206 patients recorded in the International Registry of Lung Metastases in the period 1945–1995, 328 (6.3%) had operations for pulmonary stage IV melanoma. The aims of the Registry, methodology of data collection and analysis have been published previously (Pastorino et al, 1997). In summary, all patients who had undergone resection of pulmonary metastases with curative intent were eligible for inclusion if their primary tumour, as well as metastases in other organs, had been effectively treated. Eighteen major centres from Europe, the USA and Canada took part in the project.

### Data analysis

Survival time was calculated from first metastasectomy to the last date of follow-up, by means of the Kaplan–Meier estimate. Mean follow-up of patients alive was 42 months.

The impact on survival of the following variables was tested: age, sex, radicality of metastasectomy, time to pulmonary metastases (time from surgery of primary melanoma to diagnosis of pulmonary metastases, TPM), number of pathologically confirmed metastases, resection volume, nodal involvement, delay of surgery from diagnosis of metastases to metastasectomy, chemotherapy, number of pulmonary metastasectomy operations per patient. Differences were tested by the log-rank test. A Cox regression model evaluated all variables with statistically significant impact on survival.

Finally, the prognostic grouping proposed by the International Registry of Lung Metastases in 1997 was tested to assess its value in patients with metastatic melanoma.

### Patient details

One hundred and eighty-one patients were male (55.2%) and 147 were female (44.8%) with a mean age of 49 years (range 14–78). Sixty-eight patients (20.7%) had adjuvant therapy in addition to surgical resection of the primary melanoma (chemotherapy with or without radiotherapy in 59 patients, radiotherapy in nine patients). Before metastasectomy, 73 patients (22.2%) required further treatment for recurrence of melanoma at the primary site (51 for single recurrence and 22 for multiple recurrence).

Mean TPM was 53 months for the whole series, 54 months for radical metastasectomies and 42 months for incomplete resections. Fifteen patients had one or more lung metastases synchronous with primary tumour, 40 patients had a TPM < 12 months, 102 had a TPM of 12–35 months, 163 patients (49.7%) had a TPM > 36 months (Table 1). Information about TPM was not available for eight patients (2.4%).

Information about the number of lesions radiologically evident was available in 235 patients: one lesion only was evident in 153 cases (65.1%), multiple lesions were evident in 82 (34.9%).

### RESULTS

### Surgery

The surgical approach was thoracotomy in 247 patients (75.3%), sternotomy in 74 (22.5%) and video-assisted thoracoscopy in seven (2.1%). The types of lung resection performed are listed in Table 1. An extrapulmonary resection (chest wall, diaphragm, pleura, lymph nodes, mediastinal structures, liver) was required in 50 patients (15.2%).

On pathological assessment, single metastases were present in 197 patients (60%) and multiple metastases in 131 (40%). Fifty-two patients had four or more metastases. Of the 153 patients with a solitary radiological lesion, 128 had only one pathologically proven metastasis (the accuracy of radiological assessment for single vs multiple lesions was 84%). Evaluation of the hilar or mediastinal nodes resulted positive in 26 patients (8%). Complete resection of all melanoma metastases was performed in 282 patients (86% radical metastasectomy group); 46 patients had a non-radical metastasectomy (14%). Four patients died in the post-operative period (1.2%). Adjuvant chemotherapy was used in 107 cases (32.6%), prior to surgery in 47 patients and post surgery in 60. Overall, 300 patients had one metastasectomy operation, 23 had two metastasectomies, and five had three metastasectomies.

### Survival

Overall actuarial survival was 18% at 5 years and 14% at 10 years (median survival 17 months). The overall actuarial survival of patients in which complete resection was achieved (R0, $n$ = 282) was 22% at 5 years and 16% at 10 years; no patient with incomplete resection (R1–2, $n$ = 46) was alive at 5 years (median survival 19 and 11 months respectively, $P$ < 0.01, Figure 1).

In the R0 group, patients with a TPM of > 36 months had a better long-term survival than patients with a TPM < 36 months (30% vs 15% at 5 years and 22% vs 11% at 10 years, $P$ < 0.01).

The number of pathologically proven metastases also affected survival: patients with a single lesion had better survival than those with multiple lesions (25% vs 19% at 5 years and 22% vs 7% at 10 years, $P$ = 0.03). Prognosis was significantly poorer with four or more metastases (8% at 5 years).

No effect on survival was demonstrated for age, sex, surgical approach, type of lung resection, extrapulmonary resection, chemotherapy before or after metastasectomy, number of metastasectomies, hilar-or mediastinal-node involvement.

Postponing metastasectomy did not aid the selection of patients with better prognosis. In fact the 5-year survival of patients operated within a month of detection (170 patients) was similar to the survival of those operated upon later (158 patients), 20% vs 26% respectively (median survival 30 vs 22 months, $P$ = 0.3).

**Table 1** Lung metastasectomies for melanoma: clinical features of the International Registry Series

| | R0 | R1–2 | Total | % |
|---|---|---|---|---|
| Mean age | 49 | 47 | 49 | – |
| Males | 156 | 25 | 181 | 55.2 |
| Delay | | | | |
| <1 month | 150 | 20 | 170 | 51.8 |
| >1 month | 132 | 26 | 158 | 48.2 |
| Approach | | | | |
| unilateral | 224 | 30 | 254 | 77.4 |
| bilateral | 58 | 16 | 74 | 22.6 |
| Resection | | | | |
| pneumonectomy | 13 | 1 | 14 | 4.3 |
| lobectomy | 76 | 8 | 84 | 25.6 |
| sublobar | 194 | 36 | 230 | 70.1 |
| TPM (months) | | | | |
| 0 | 12 | 3 | 15 | 4.5 |
| 1–11 | 30 | 10 | 40 | 12.2 |
| 12–35 | 89 | 13 | 102 | 31.1 |
| >36 | 143 | 20 | 163 | 49.7 |

(R0 = Radical metastasectomy; R1–2 = non-radical metastasectomy; delay = time from diagnosis to operation; TPM = time to pulmonary metastases)

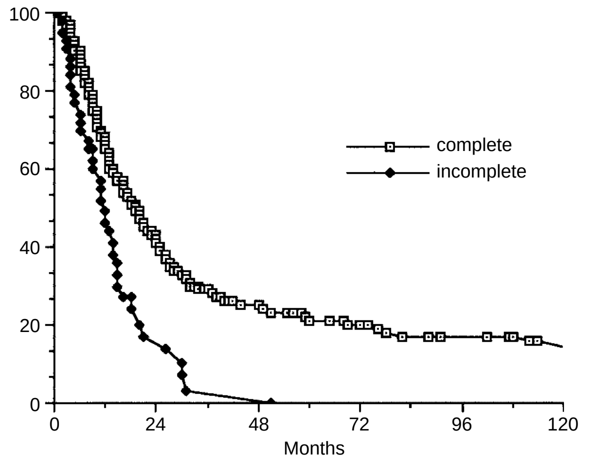

**Figure 1** Long = term survival according to the radicality of metastasectomy (16% vs 0% at 10 years, $P$ < 0.01)

© 2000 Cancer Research Campaign

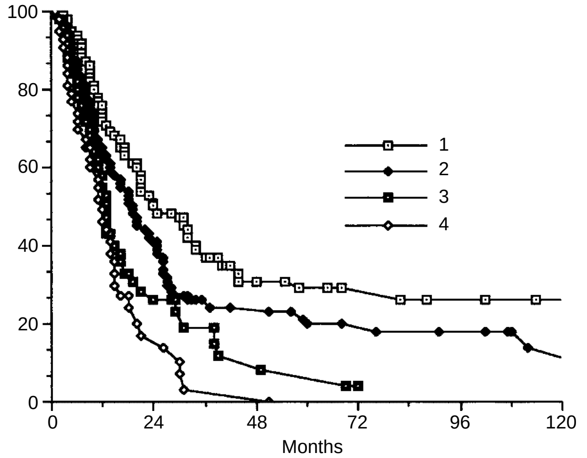

**Figure 2** Prognostic grouping proposed by the Registry in 1997 confirmed its value when applied to lung metastases from melanoma. Benefit of metastasectomy in Group I and II patients is still evident 10 years after metastasectomy

### Pattern of failure

During the follow-up period, 221 patients died (crude mortality rate 67%), 184 in the R0 group (crude mortality rate 65%) and 37 in the R1–2 group (mortality rate 80%). Mean time from metastasectomy to death was 18 months (19 months for R0 patients and 9 months for R1–2 patients). One hundred and eighty patients (63.8%) experienced recurrence of melanoma after radical pulmonary metastasectomy: intrathoracic recurrence in 48 cases, intra- and extrathoracic in 50 cases and extrathoracic relapse alone in 82 cases. The most common sites of extrathoracic disease were brain (58 patients) and liver (15 patients).

Of 48 patients with intrathoracic recurrence, further surgery was possible in 28 (58.3%). Twenty-three such patients had a second metastasectomy, five had three metastasectomies. Their long-term survival was 19% at 5 years.

### Prognostic grouping

The three significant prognostic factors from multivariate analysis (radicality, TPM, number of lesions) were used to design a system of classification according to the proposal of the International Registry of Lung Metastases (Pastorino et al, 1997). Following these guidelines, the 328 patients of the study were divided into four groups:

- Group I: patients who had radical metastasectomy and no adverse prognostic factors (TPM > 36 months and single metastasis, $n$ = 96)
- Group II: radical metastasectomy and one unfavourable factor (TPM < 36 months or multiple metastases, $n$ = 126)
- Group III: radical metastasectomy and two unfavourable factors (TPM < 36 months and multiple metastases, $n$ = 52)
- Group IV: incomplete metastasectomy ($n$ = 46).

The survival difference among these groups was highly significant (log-rank 2 29.6, df 3, Figure 2). Group I survival was 29% at 5 years and 26% at 10 years. Group II survival was 20% at 5 years and 11% at 10 years. Group III survival was 7% at 5 years. Group IV had no 5-year survivors.

### DISCUSSION

Metastatic melanoma has a very poor prognosis, with a median survival of about 9 months (Falkson, 1998). For some of these patients with limited metastatic disease, surgical resection can offer the best chance of cure (Fletcher, 1998). This was confirmed even in the subset of pulmonary metastatic melanoma in two large series previously published (Tafra et al, 1995; Harpole et al, 1992). However, pulmonary metastasectomy for melanoma has the lowest long-term survival after surgery compared to germ cell tumours, carcinomas and sarcomas (Pastorino et al, 1997). This peculiarity emphasizes the importance of prognostic indicators to avoid surgery in patients who will not benefit. When the lung is the only site of recurrence, radicality of pulmonary metastasectomy is the main factor affecting survival (Harpole et al, 1992; Tafra et al, 1995; Pastorino et al, 1997).

We tested many variables on the IRLM series, which represents the largest group of patients with surgically treated pulmonary metastatic melanoma available in the literature, in the search for independent prognostic predictors after lung metastasectomy. In particular, the grouping system proposed by the International Registry of Lung Metastases in 1997 was tested, taking into account the radicality of resection and two more risk factors: number of pathologically proven lesions and time elapsed from primary tumour to pulmonary metastases.

Having a single lesion and a long time to pulmonary metastases means a better survival in surgically treated patients with pulmonary metastases from melanoma (Harpole et al, 1992); it was suggested by Tafra et al (1995) and is confirmed by our study. Applying the IRLM grouping system, long-term results remain satisfactory at 10 years when a radical resection is achieved and only one risk factor is present (Groups I and II). On the contrary, metastasectomy is not effective when multiple metastases are associated with a TPM shorter than 3 years.

Given the fact that the proposed grouping system has a good prognostic value when applied retrospectively, it would seem logical to use a similar system to select patients with pulmonary metastases from melanoma for surgery.

Preoperative evaluation of the number of lesions is crucial. In the Registry experience, the accuracy of radiological assessment in detection of single lesions was good (84%). CT scan was not routinely used in the early period of the series but accuracy remains very similar throughout (considering only the last 10 years, period 1985–1995: accuracy 86%). It is expected that spiral CT scan and possibly PET will improve the diagnostic power of radiological assessment (Holder et al, 1998). Applying the IRLM grouping system prospectively, it can be argued that limiting surgery for pulmonary metastases to Groups I and II would produce better results in melanoma patients.

Other factors indicated in the literature as important prognostic indicators were considered in this study. Chemotherapy, which is reportedly associated with better survival in pulmonary metastatic melanoma (Harpole et al, 1992), did not influence survival after surgery in the IRLM series. This conclusion is greatly limited by the retrospective nature of the IRLM data because information on inclusion criteria, regimen used and response rates are missing. Moreover, the role of immunotherapy, reported to be effective as a post-surgery adjuvant treatment (Tafra et al, 1995), was not verified by this study for the same reason.

*© 2000 Cancer Research Campaign*

The importance of nodal spread in metastatic disease cannot be disregarded. Harpole described a poorer prognosis for node-positive patients. This point was not confirmed by our study. This difference could be due to at least two factors: the small number of node-positive patients in the IRLM series (26 patients) compared with Harpole's series (507); all patients with clinical node-positivity were compared with node-negative cases in Harpole's series, regardless of whether they had been resected or not. The IRLM series considered only resected patients and it is reasonable to assume that patients with clinical mediastinal node-positivity were excluded from operation. Moreover, lymphadenectomy is not routinely performed during lung metastasectomy and therefore some patients may not be properly staged.

Recently, tumour doubling time (TDT) was indicated as an important tool for selection of patients with melanoma pulmonary metastases who are likely to benefit from metastasectomy (Ollila et al, 1998). For a TDT < 60 days, chemotherapy and biological therapy are recommended with surgery performed afterwards only if the TDT has been modified. Unfortunately, no data about TDT was available from the Registry. TPM and TDT express both the biological behaviour of the disease before and after clinical detection of pulmonary metastases. If TDT is shown to have an impact independent from TPM, it could be tested to identify a subset of patients with short TPM but long TDT who might still have a chance of cure after surgery.

In conclusion, despite previously cited limitations, data from the Registry confirms that surgery plays an important role in carefully selected cases of pulmonary metastatic melanoma. The grouping system proposed by the IRLM can help this selection, as Groups I and II maintain an acceptable survival even 10 years after metastasectomy. However, patients with multiple metastases and a short TPM have an unfavourable outcome that is not improved by surgery.

### REFERENCES

Balch CM and Milton GW (1985) Diagnosis of metastatic melanoma at distant sites. In *Cutaneous melanoma: clinical management and treatment results worldwide*, Balch CM, Milton GW (eds) pp 221. Philadelphia: Lippincott

Cahan WG (1973) Excision of melanoma metastases to lung: problems in diagnosis and management. *Ann Surg* **178**: 703–709

Falkson CI, Ibrahim J, Kirkwood JM, Coates AS, Atkins MB and Blum RH (1998) Phase III trial of dacarbazine with interferon alpha-2b versus dacarbazine with tamoxifen versus dacarbazine with interferon alpha-2b and tamoxifen in patients with metastatic malignant melanoma: an Eastern Cooperative Oncology Group study. *J Clin Oncol* **16**: 1743–1751

Fletcher WS, Pommier RF, Lum S and Wilmarth TJ (1998) Surgical treatment of metastatic melanoma. *Am J Surg* **175**: 413–417

Gorenstein LA, Putnam JB, Natarajan, Balch CA and Roth JA (1991) Improved survival after resection of pulmonary metastases from malignant melanoma. *Ann Thorac Surg* **52**: 204–210

Harpole DH, Johnson CM, Wolfe WG, George SL and Seigler HF (1992) Analysis of 945 cases of pulmonary metastatic melanoma. *J Thorac Cardiovasc Surg* **103**: 743–750

Holder WD, White RL, Zuger JH, Easton EJ and Greene FL (1998) Effectiveness of positron emission tomography for the detection of melanoma metastases. *Ann Surg* **227**: 764–771

Karakousis CP, Velez A, Driscoll DL and Takita H (1994) Metastasectomy in malignant melanoma. *Surgery* **115**: 295–302

Ketcham AS and Balch CM (1985) Classification and staging system. In *Cutaneous melanoma: clinical management and treatment results worldwide*, Balch CM and Milton GW (eds) pp 55–62. Philadelphia: Lippincott

Mathisen DJ, Flye MW and Peabody J (1979) The role of thoracotomy in the management of pulmonary metastases from malignant melanoma. *Ann Thorac Surg* **27**: 295–299

Mountain CF, McMuntrey MJ and Hermes KE (1984) Surgery for pulmonary metastasis: a 20 years experience. *Ann Thorac Surg* **38**: 323–330

Olilla DW, Stern SL and Morton DL (1998) Tumour doubling time: a selection factor for pulmonary resection of metastatic melanoma. *J Surg Oncol* **69**: 206–211

Pastorino U, Buyse M, Friedel G, Ginsberg RJ, Girard P, Goldstraw P, Johnston M, McCormack P, Pass H and Putnam JB (1997). Long-term results of lung metastasectomy: prognostic analysis based on 5206 cases. *J Thorac Cardiovasc Surg* **113**: 37–49

Pogrebniak HW, Stovroff M, Roth JA and Pass HI (1988) Resection of pulmonary metastases from malignant melanoma: results of 16-years experience. *Ann Thorac Surg* **46**: 20–23

Slingluff CL, Dodge RK, Stanley WE and Seigler HF (1992) The annual risk of melanoma progression. *Cancer* **70**: 1917–1927

Tafra L, Dale PS, Wanek LA, Ramming KP and Morton DL (1995) Resection and adjuvant immunotherapy for melanoma metastatic to the lung and thorax. *J Thorac Cardiovasc Surg* **110**: 119–128

Thayer JO and Overholt RH (1985) Metastatic melanoma to the lung: long term results of surgical excision. *Am J Surg* **149**: 558–562

Wong JH, Skinner KA, Kim KA, Foshag LJ and Morton DL (1993) The role of surgery in the treatment of nonregionally recurrent melanoma. *Surgery* **113**: 389–394

*© 2000 Cancer Research Campaign*
